# Polygenic risk, familial liability and stress reactivity in psychosis: an experience sampling study

**DOI:** 10.1017/S0033291721004761

**Published:** 2023-05

**Authors:** Anita Schick, Ruud van Winkel, Bochao D. Lin, Jurjen J. Luykx, Sonja M.C. de Zwarte, Kristel R. van Eijk, GROUP Investigators, Inez Myin-Germeys, Ulrich Reininghaus

**Affiliations:** 1 Department of Public Mental Health, Central Institute of Mental Health, Medical Faculty Mannheim, Heidelberg University, Mannheim, Germany; 2 KU Leuven, Department of Neuroscience, Research Group Psychiatry, Center for Clinical Psychiatry, Leuven, Belgium; 3 Department of Translational Neuroscience, UMC Brain Center, University Medical Center Utrecht, Utrecht University, Utrecht, The Netherlands; 4 Department of Psychiatry, UMC Brain Center, University Medical Center Utrecht, Utrecht University, Utrecht, The Netherlands; 5 Second Opinion Outpatient Clinic, GGNet, Warnsveld, The Netherlands; 6 Department of Neurology and Neurosurgery, University Medical Center Utrecht Brain Center, Utrecht University, Utrecht, The Netherlands; 7 KU Leuven, Department of Neuroscience, Research Group Psychiatry, Center for Contextual Psychiatry, Leuven, Belgium; 8 Department of Psychiatry and Neuropsychology, School for Mental Health and Neuroscience, Maastricht University, Maastricht, The Netherlands; 9 ESRC Centre for Society and Mental Health, King's College London, London, UK; 10 Center for Epidemiology and Public Health, Health Service and Population Research Department, Institute of Psychiatry, Psychology & Neuroscience, King's College London, London, UK

**Keywords:** Digital phenotyping, ecological momentary assessment, gene–environment interaction, polygenic risk, stress sensitivity

## Abstract

**Background:**

There is evidence for a polygenic contribution to psychosis. One targetable mechanism through which polygenic variation may impact on individuals and interact with the social environment is stress sensitization, characterized by elevated reactivity to minor stressors in daily life. The current study aimed to investigate whether stress reactivity is modified by polygenic risk score for schizophrenia (PRS) in cases with enduring non-affective psychotic disorder, first-degree relatives of cases, and controls.

**Methods:**

We used the experience sampling method to assess minor stressors, negative affect, positive affect and psychotic experiences in 96 cases, 79 first-degree relatives, i.e. siblings, and 73 controls at wave 3 of the Dutch Genetic Risk and Outcome of Psychosis (GROUP) study. Genome-wide data were collected at baseline to calculate PRS.

**Results:**

We found that associations of momentary stress with psychotic experiences, but not with negative and positive affect, were modified by PRS and group (all *p*_FWE_<0.001). In contrast to our hypotheses, siblings with high PRS reported less intense psychotic experiences in response to momentary stress compared to siblings with low PRS. No differences in magnitude of these associations were observed in cases with high *v.* low level of PRS. By contrast, controls with high PRS showed more intense psychotic experiences in response to stress compared to those with low PRS.

**Conclusions:**

This tentatively suggests that polygenic risk may operate in different ways than previously assumed and amplify reactivity to stress in unaffected individuals but operate as a resilience factor in relatives by attenuating their stress reactivity.

## Introduction

Recent years have shown significant advances through large-scale collaboration in genome-wide association studies (GWAS), which have generated replicated findings on a number of common risk alleles and copy number variants, suggesting that the risk of psychosis is polygenic (Lee et al., 2019; McGrath, Mortensen, Visscher, & Wray, 2013; Schizophrenia Working Group of the Psychiatric Genomics et al., 2014). Individuals that carry a higher number of risk variants have a higher risk for developing psychotic disorder. Further, there is consistent evidence from numerous twin and family studies that the risk for developing a psychotic disorder is increased in first-degree relatives of patients with the disorder (Guloksuz et al., 2019; van Os, Reininghaus, & Meyer-Lindenberg, 2017a), which suggests a familial liability to psychosis (Islam et al., 2017). Familial liability may derive from a shared environment, i.e. the shared exposure to social adversity (e.g. bereavement). In addition, the environment of children is strongly influenced by their parents or their parents’ genes (Rutter, Moffitt, & Caspi, 2006; van Os, Rutten, & Poulton, 2008). These findings broadly support a liability-threshold model.

Evidence further suggests that socio-environmental factors play an important role in the development of psychosis (Heinz, Deserno, & Reininghaus, 2013; Klippel et al., 2017; Morgan, Charalambides, Hutchinson, & Murray, 2010; Rauschenberg et al., 2017; Reininghaus et al., 2016b, 2016c; Uher, 2014; van Os & Rutten, 2014; Waszczuk et al., 2020). One targetable mechanism through which polygenic variation has been posited to impact on individuals and interact with the social environment to increase the risk for psychosis is stress sensitization (Collip, Myin-Germeys, & Van Os, 2008; Rauschenberg et al., 2017; Reininghaus et al., 2016b, 2016c). The proposition here is that the stress response is amplified in individuals with increased polygenic risk, such that they experience a greater response to even minor stressors in daily life. This process of sensitization operates at various levels of causation. At the behavioural level, the most commonly used marker of this underlying process is stress reactivity characterized by (i) stronger emotional reactions, and (ii) more intense psychotic experiences in response to minor stressors in daily life (Klippel et al., 2017; Myin-Germeys & van Os, 2007; Myin-Germeys, van Os, Schwartz, Stone, & Delespaul, 2001; Rauschenberg et al., 2017; Reininghaus et al., 2016c). Minor stressors, i.e. unpleasant events, activities and social situations, as well as emotional reactions and psychotic experiences may arguably be best measured using the experience sampling method (ESM; Csikszentmihalyi & Larson, 1987; Klippel et al., 2017; Myin-Germeys et al., 2018; Rauschenberg et al., 2017; Reininghaus, 2018; Reininghaus, Depp, and Myin-Germeys, 2016a; Reininghaus et al., 2016c), a structured digital intensive longitudinal data collection technique that allows to assess moment-to-moment variation in daily life. Using this method, Myin-Germeys et al. (2001) were the first to show a gradient in stress reactivity that paralleled the level of familial liability, in that stress reactivity was elevated in cases with psychotic disorder and first-degree relatives compared with controls. Recent advances in GWAS allow to investigate this further using a molecular genetic measure of polygenic risk, i.e. polygenic risk scores (PRS), that may modify individuals' response to minor stressors. Recently, a first experience sampling study did not find evidence on stress reactivity being modified by PRS in a sample of young healthy adults (Pries et al., 2020). However, previous studies have not investigated this in cases with psychotic disorder and their first-degree relatives to elucidate whether stress reactivity operates in individuals with increased familial liability, and is modified by polygenic risk, in pathways to psychosis. Furthering our understanding of this targetable mechanism is relevant as a basis for developing effective interventions.

In the current study, we aimed to investigate whether the associations of momentary stress with (i) negative affect, (ii) positive affect and (iii) psychotic experiences are modified by PRS and liability to psychosis in cases, siblings and controls. Specifically, we aimed to test the following hypotheses: (1) within each group, the magnitude of associations of momentary stress with (i) negative affect, (ii) positive affect and (iii) psychotic experiences is greater in individuals with high PRS *v.* individuals with low PRS (H1); and (2) the difference in magnitude of associations of momentary stress with (i) negative affect, (ii) positive affect and (iii) psychotic experiences (i.e. the difference in responses to stress) between those with high *v.* low levels of PRS is greater in (a) cases than in controls, (b) siblings than in controls and (c) cases than in siblings (H2).

## Methods

### Participants

Data were collected as part of a longitudinal study, the Genetic Risk and Outcome of Psychosis Project (GROUP) in the Netherlands and Belgium (Korver, Quee, Boos, Simons, & de Haan, 2012). Inclusion criteria for cases were the age between 16 and 50 years, meeting full DSM-IV criteria for a non-affective psychotic disorder and estimated level of intelligence above 70. Siblings of cases were recruited via the participating cases and included when they were aged 16–50 years. Controls were contacted through mailing lists. Inclusion criteria for controls were the age between 16 and 50 years, no lifetime psychotic disorder and no first-degree family member with a lifetime psychotic disorder. The Positive and Negative Syndrome Scale (PANSS; Kay, Fiszbein, & Opler, 1987) and a short version of the Wechsler Adult Intelligence Scale (WAIS-III; Wechsler, 1977) were administered to all participants in order to assess clinical symptoms and intellectual abilities. Further, all participants completed the social functioning scale (Birchwood, Smith, Cochrane, Wetton, & Copestake, 1990). Detailed information on sample characteristics and recruitment methods has been previously described (Korver et al., 2012).

### Genotyping, imputation and polygenic risk score (PRS)

Genotype data for 2812 individuals was generated on a customized Illumina array with 570 038 SNPs. Quality control (QC) procedures were performed using PLINK v1.9 (Purcell et al., 2007) (see online Supplementary Material). In total, 2505 individuals passed the QC steps. Then, SNPs were imputed on the Michigan server (Das et al., 2016). After post-imputation QC, PRS were calculated for 2505 samples using schizophrenia-associated alleles and effect sizes reported in the GWAS summary statistics from the Psychiatric Genetics Consortium Schizophrenia Workgroup freeze 2 (PGC-2; Schizophrenia Working Group of the Psychiatric Genomics et al., 2014). To prevent potential overlap in study population to impact our results, all Dutch and Belgian individuals had been excluded from the PGC-2 GWAS to allow unbiased PRS computation. PRS were calculated using PLINK's score function for 12 GWAS *p* value thresholds. The PRS *p*_t_ = 0.05 explained most of the variance of schizophrenia case-control status (see online Supplementary Material). Hence, it was selected to perform the following regression analyses.

### ESM measures

Using ESM, momentary stress, affect and psychotic experiences were assessed over the course of six consecutive days. There is good evidence on the feasibility of ESM, especially with respect to patient samples (Myin-Germeys et al., 2018, Oorschot et al., 2009). Participants received a dedicated digital device (i.e. the PsyMate, www.psymate.eu/) and were asked to complete 10 ESM assessments per day. ESM data were only used if participants completed more than 20 assessments in total (Myin-Germeys et al., 2001; Reininghaus et al., 2016c). A detailed description of the ESM measures is shown in Table 1.

**Table 1.** ESM measures

Measure	ESM items
Momentary stress	
Event-related stress	Participants rated the most important event that happened in the time since the last assessment on a 7-point Likert scale (−3 = very unpleasant, 0 = neutral, 3 = very pleasant). Responses were recoded such that higher scores reflect greater event-related stress. The scores −3 to −1 were then set to missing.
Activity-related stress	Activity-related stress was assessed by asking participants to report their current activity and then judge their competences and volition (‘I am not skilled to do this activity’, ‘I would rather do something else’ and ‘This activity requires effort’) using 7-point Likert scales. The mean of these three items indicated activity-related stress.
Social stress	Social stress was measured by asking participants to evaluate the social context either when other people were present or when they were alone by answering the items ‘I feel at ease’/‘I enjoy being alone’ and ‘I would rather be alone’/‘I would prefer to have company’ using two 7-point Likert scales.
Composite stress	A mean score of event-related, activity-related and social stress was calculated in line with Pries et al. (2020), Rauschenberg et al. ( 2021c).
Affect	
Negative affect	Negative affect was assessed by five items (anxiousness, loneliness, insecurity, irritation and feeling down) using 7-point Likert scales ranging from ‘not at all’ (rating of 1) to ‘very much’ (rating of 7). Internal consistency of negative affect items within individuals was satisfying (Cronbach's *α* = 0.62). The mean of the negative affect items was calculated as overall measure of negative affect.
Positive affect	Positive affect was assessed by four items (cheerfulness, satisfaction, enthusiasm and feeling relaxed) using 7-point Likert scales ranging from ‘not at all’ (rating of 1) to ‘very much’ (rating of 7). We found satisfying internal consistency within individuals (Cronbach's *α* = 0.78) for positive affect. The mean of the positive affect items was calculated as overall measure of positive affect.
Psychotic experiences	Psychotic experiences were assessed using eight items on thought problems and hallucinations (‘I see things that aren't really there’, ‘I hear things that aren't really there’, ‘I feel suspicious/paranoid’, ‘I feel unreal’, ‘My thoughts are influenced by others’, ‘I can't get these thoughts out of my head’, ’I cannot express my thoughts’, ‘I feel like I am losing control’) rated on 7-point Likert scales ranging from ‘not at all’ (rating of 1) to ‘very much’ (rating of 7) as previously described (Myin-Germeys et al., 2001; Reininghaus et al., 2016c). The ESM items for psychotic experiences showed satisfying internal consistency within individuals (Cronbach's *α* = 0.64).
Burden of assessment	The item ‘This beep was disturbing’ was rated at the end of each assessment on a 7-point Likert scale from ‘not at all’ (rating of 1) to ‘very much’ (rating of 7).

*Note:* The experience sampling methodology (ESM) was used and prompted participants 10 times a day on 6 consecutive days with a semi-random sampling scheme within a fixed, predefined time frame.

### Statistical analysis

For the current analysis, the GROUP wave 3 data set with release number 7.0 and ESM data set with release number 2.0 were used. All analyses were carried out using STATA version 15.1 (StataCorp., 2017). Pairwise group comparisons were performed regarding basic group characteristics and the mean of the independent and dependent variables using one-way analysis of variance. We then fitted linear mixed models to account for the multilevel structure of ESM data using the mixed command in STATA. For each momentary stressor (composite stress, event-related, activity-related, social stress), a separate model was fitted with (i) negative affect, (ii) positive affect and (iii) psychotic experiences as outcome variables, while controlling for potential confounders (i.e. age, gender, IQ and the first three principal components to control for genetic ancestry). We added two-way (momentary stress × PRS) and three-way (momentary stress × PRS × group) interactions to test whether associations between momentary stress and (i) negative affect, (ii) positive affect and (iii) psychotic experiences were modified by PRS and group (cases, siblings, controls). In addition, multilevel mixed tobit regression models (Tobin, 1958) were fitted to account for skewness in data on psychotic experiences (see online Supplementary Table S3).

We used Wald tests to assess the statistical significance of each interaction term. To investigate whether associations of each momentary stressor with (i) negative affect, (ii) positive affect or (iii) psychotic experiences were greater in individuals with high *v.* low PRS, continuous independent variables were standardized (mean = 0, s.d. = 1) for interpreting significant interaction terms and examining the difference in associations between high (mean + 1 s.d.), and low (mean − 1 s.d.) PRS within and across groups (cases, siblings, controls) (Aiken & West, 1991; Cohen, Cohen, West, & Aiken, 2003). Specifically, we calculated linear combinations of coefficients using the lincom command in STATA testing the hypotheses that: (1) within each group, the magnitude of associations of each momentary stressor with (i) negative affect, (ii) positive affect and (iii) psychotic experiences was greater in individuals with high *v.* low PRS (mean ± 1 s.d. of continuous PRS) (Aiken & West, 1991; Cohen et al., 2003) (H1); and (2) the difference in magnitude of associations of each momentary stressor with (i) negative affect, (ii) positive affect and (iii) psychotic experiences in those individuals with high *v.* low PRS (mean ± 1 s.d. of continuous PRS) was greater in (a) cases than in controls, (b) relatives than in controls, and (c) cases than in relatives (H2). Likelihood ratio tests were used to evaluate improvement in model fit. We adjusted the significance level of likelihood ratio tests for the three-way interactions in order to correct for Type-1 error proliferation using family-wise error correction (*p*_FWE_ values). The unadjusted *p* value was multiplied by the total number of tests, i.e. by 12 (four stress measures, three groups). Two-tailed *p*_FWE_ < 0.05 was considered nominally statistically significant. We standardized continuous ESM and PRS variables (mean = 0, s.d. = 1) for interpreting significant three-way interactions.

## Results

### Sample characteristics

The full GROUP sample consisted of 3684 participants and ESM was completed at wave 3. The analytic sample with available ESM and PRS data comprised 248 participants, i.e. 96 cases with non-affective psychosis, 79 siblings of cases and 73 controls. As the prevalence of risk alleles varies across ethnic groups, the analytic sample was selected to comprise participants of White European decent only. There were notable differences in sociodemographic and clinical characteristics of the analytic sample compared to the full GROUP sample of cases, siblings and controls (see online Supplementary Table S2).

Within the analytic sample, cases, siblings and controls differed in socio-demographic and clinical characteristics as shown in Table 2. Specifically, cases were, on average, younger (*β* = −1.81, 95% CI −2.243 to −1.371, *p* < 0.001), had lower IQ estimates (*β* = −10.94, 95% CI −11.74 to −10.13, *p* < 0.001) and comprised more men (*β* = −0.28, 95% CI −0.30 to −0.26, *p* < 0.001) than siblings. Furthermore, cases showed reduced social functioning (*β* = −9.32, 95% CI −11.32 to −7.31, *p* < 0.001) and increased positive (*β* = 4.65, 95% CI 3.72–5.58, *p* < 0.001) as well as negative symptoms (*β* = 3.61, 95% CI 2.78–4.45, *p* < 0.001) compared to siblings. The same differences were evident in cases *v.* controls (all *p* < 0.001, see Table 2). However, PRS was higher in cases than in siblings (*β* = 3.46, 95% CI 3.83–3.10, *p* < 0.001) and controls (*β* = 3.07, 95% CI 2.71–3.45, *p* < 0.001). There was some evidence that PRS in siblings was, on average, lower compared to controls (*β* = −0.39, 95% CI −0.77 to −0.002, *p* = 0.05). In addition, siblings and controls did not differ in social functioning (*β* = −1.18, 95% CI −3.33 to 0.98, *p* = 0.28) or symptoms (PANSS positive symptoms: *β* = −0.09, 95% CI −1.08 to 0.90, *p* = 0.86; PANSS negative symptoms: *β* = −0.16, 95% CI −0.73 to 1.05, *p* = 0.73).

**Table 2.** Sample characteristics of cases, siblings and controls

	Cases (*N* = 96)	Siblings (*N* = 79)	Controls (*N* = 73)	Test statistics		Cases *v*. controls		Cases *v*. siblings		Siblings *v*. controls	
				*F* (df)	*p*	adj. B (95% CI)	*p*	adj. B (95% CI)	*p*	adj. B (95% CI)	*p*
Age (years), mean (s.d.)	33.45 (7.54)	35.26 (8.68)	40.50 (11.55)	513.97 (2)	<0.001	−7.05 (−7.49 to −6.61)	<0.001	−1.81 (−2.24 to −1.37)	<0.001	−5.24 (−5.70 to −4.78)	<0.001
Gender, *n* (%)				706.57 (2)	<0.001	−0.41 (−0.43 to −0.38)	<0.001	−0.28 (−0.30 to −0.26)	<0.001	−0.13 (−0.15 to −0.10)	<0.001
Male	63 (65.62)	29 (36.71)	18 (24.66)								
Female	33 (33.38)	50 (63.29)	55 (75.34)								
IQ estimate, mean (s.d.)	101.37 (16.77)	112.30 (16.44)	114.71 (17.86)	609.18 (2)	<0.001	−13.34 (−14.15 to −12.53)	<0.001	−10.94 (−11.74 to −10.13)	<0.001	−2.41 (−3.43 to −1.57)	<0.001
Clinical characteristics, mean (s.d.)											
Social functioning	114.10 (8.01)	123.42 (6.22)	124.60 (5.14)	64.16 (2)	<0.001	−10.49 (−12.56 to −8.43)	<0.001	−9.32 (−11.32 to −7.31)	<0.001	−1.18 (−3.33 to 0.98)	0.28
PANSS positive	11.83 (4.72)	7.18 (0.77)	7.27 (0.58)	66.84 (2)	<0.001	4.56 (3.63 to 5.49)	<0.001	4.65 (3.72 to 5.58)	<0.001	−0.09 (−1.08 to 0.90)	0.86
PANSS negative	11.85 (4.23)	8.24 (0.92)	8.08 (0.44)	52.97 (2)	<0.001	3.77 (2.93 to 4.61)	<0.001	3.61 (2.78 to 4.45)	<0.001	0.16 (−0.73 to 1.05)	0.73
PRS, mean (s.d.)	−1.34 (7.71)	−4.81 (7.03)	−4.42 (8.65)	211.20 (2)	<0.001	3.07 (2.71 to 3.45)	<0.001	3.46 (3.83 to 3.10)	<0.001	−0.39 (−0.77 to −0.002)	0.05

PRS, polygenic risk score; PANSS, Positive and Negative Syndrome Scale; s.d., standard deviation; *v*., versus; CI, confidence interval.

As shown in Table 3, cases completed fewer ESM assessments (i.e. beeps) compared to siblings (*β* = −0.63, 95% CI −1.07 to −0.18, *p* = 0.006) and controls (*β* = −1.50, 95% CI −2.00 to −1.01, *p* < 0.001), whereas siblings completed fewer assessments than controls (*β* = −1.78, 95% CI 2.21 to −1.34, *p* < 0.001). There were no differences between cases and controls (*β* = 0.07, 95% CI −0.06 to 0.20, *p* = 0.31) in interference of ESM assessments with their daily life. However, interference of ESM assessment with daily life was lower in cases than in siblings (*β* = −0.50, 95% CI −0.62 to −0.39, *p* < 0.001) and higher in siblings than in controls (*β* = 0.57, 95% CI 0.46–0.69, *p* < 0.001). Cases reported, on average, higher momentary stress (i.e. composite stress, event-related stress, activity-related stress and social stress) compared to siblings (e.g. composite stress: *β* = 0.30, 95% CI 0.25–0.35, *p* < 0.001) and controls (e.g. composite stress: *β* = 0.51, 95% CI 0.46–0.57, *p* < 0.001). Further, cases reported higher negative affect than both siblings (*β* = 0.68, 95% CI 0.64–0.72, *p* < 0.001) and controls (*β* = 0.71, 95% CI 0.66–0.76, *p* < 0.001), as well as lower positive affect than siblings (*β* = −0.41, 95% CI −0.47 to −0.35, *p* < 0.001) and controls (*β* = −0.52, 95% CI −0.59 to −0.45, *p* < 0.001). Intensity of psychotic experiences were, on average, greater in cases compared to both siblings (*β* = 0.39, 95% CI 0.36–0.42, *p* < 0.001) and controls (*β* = 0.39, 95% CI 0.36–0.42, *p* < 0.001). Although siblings and controls differed in some momentary stress measures (e.g. composite stress: *β* = 0.21, 95% CI 0.17–0.26, *p* < 0.001) and positive affect (*β* = −0.11, 95% CI −0.17 to −0.05, *p* < 0.001), this was not the case for negative affect (*β* = 0.03, 95% CI −0.01 to 0.08, *p* = 0.16) or psychotic experiences (*β* = −0.002, 95% CI −0.03 to 0.02, *p* = 0.88).

**Table 3.** Aggregate ESM scores for momentary stress, negative affect, positive affect and psychotic experiences in cases, siblings and controls

	Cases	Siblings	Controls	Test statistics		Cases *v*. controls^a^		Cases *v*. siblings^a^		Siblings *v*. controls^a^	
	Mean (s.d.)	Mean (s.d.)	Mean (s.d.)	*F* (df)	*p*	adj. β (95% CI)	*p*	adj. β (95% CI)	*p*	adj. β(95% CI)	*p*
Number of valid beeps	41.07 (9.20)	42.33 (8.96)	44.11 (8.20)	102.46 (2)	<0.001	−1.50 (−2.00 to −1.01)	<0.001	−0.63 (−1.07 to −0.18)	0.006	−0.87 (−1.31 to −0.43)	<0.001
Interference	3.00 (1.90)	3.44 (2.00)	2.87 (1.95)	60.40 (2)	<0.001	0.07 (−0.06 to 0.20)	0.31	−0.50 (−0.62 to −0.39)	<0.001	0.57 (0.46 to 0.69)	<0.001
Composite stress measure	2.14 (1.05)	1.83 (0.90)	1.62 (0.92)	250.43 (2)	<0.001	0.51 (0.46 to 0.57)	<0.001	0.30 (0.25 to 0.35)	<0.001	0.21 (0.17 to 0.26)	<0.001
Event-related stress	0.59 (0.95)	0.44 (0.80)	0.46 (0.88)	11.97 (2)	<0.001	0.14 (0.06 to 0.22)	<0.001	0.16 (0.09 to 0.23)	<0.001	−0.02 (−0.09 to 0.05)	0.65
Activity-related stress	2.65 (1.19)	2.56 (1.13)	2.24 (1.04)	122.07 (2)	<0.001	0.39 (0.33 to 0.46)	<0.001	0.06 (0.02 to 0.13)	0.011	0.31 (0.26 to 0.37)	<0.001
Social stress	2.22 (1.72)	1.66 (1.38)	1.45 (1.43)	237.59 (2)	<0.001	0.73 (0.64 to 0.82)	<0.001	0.52 (0.44 to 0.60)	<0.001	0.21 (0.13 to 0.29)	<0.001
Negative affect	2.09 (1.03)	1.44 (0.75)	1.42 (0.73)	687.51 (2)	<0.001	0.71 (0.66 to 0.76)	<0.001	0.68 (0.64 to 0.72)	<0.001	0.03 (−0.01 to 0.08)	0.16
Positive affect	4.47 (1.28)	4.82 (1.07)	4.94 (1.13)	155.48 (2)	<0.001	−0.52 (−0.56 to −0.45)	<0.001	−0.41 (−0.47 to −0.35)	<0.001	−0.11 (−0.17 to −0.05)	<0.001
Psychotic experience	1.51 (0.66)	1.11 (0.33)	1.12 (0.35)	791.28 (2)	<0.001	0.39 (0.36 to 0.42)	<0.001	0.39 (0.37 to 0.42)	<0.001	−0.002 (−0.03 to 0.02)	0.88

s.d., standard deviation; *v*., versus; CI, confidence interval.

a Adjusted for age, gender and IQ.

### Stress reactivity by PRS in cases, siblings and controls

As can be seen in Table 4, we found no evidence that the association of composite momentary stress, event-related-stress, activity-related stress and social stress, on the one hand, with (i) negative affect and (ii) positive affect, on the other, was modified by PRS in cases, siblings and controls. However, there was strong evidence for three-way interaction effects of composite momentary stress × PRS × group (*χ*^2^ = 19.70, *p*_FWE_ = 0.001), activity-related stress × PRS × group (*χ*^2^ = 22.07, *p*_FWE_ < 0.001) and social stress × PRS × group (*χ*^2^ = 17.89, *p*_FWE_ = 0.001) on (iii) psychotic experiences (see Table 5). This indicated that the associations of composite momentary stress, activity-related stress, and social stress with (iii) psychotic experiences differed between individuals with high and low levels of PRS within (H1) and across groups (H2), as detailed below.

**Table 4.** Associations of momentary stress with negative affect and positive affect in cases, siblings and controls^a^

	Cases		Siblings		Controls		LR test for interaction^b^		
	adj. *β* (95% CI)	*p*	adj. *β* (95% CI)	*p*	adj. *β* (95% CI)	*p*	χ^2^ (df)	*p*	*p*_FWE_
Outcome: negative affect									
Composite stress × PRS × group							3.91 (2)	0.14	1.00
Event-related stress × PRS × group							0.46 (2)	0.79	1.00
Activity-related stress × PRS × group							7.67 (2)	0.02	1.00
Social stress × PRS × group							4.31 (2)	0.12	1.00
Outcome: positive affect									
Composite stress × PRS × group							0.63 (2)	0.91	1.00
Event-related stress × PRS × group							0.1 (2)	0.95	1.00
Activity-related stress × PRS × group							1.0 (2)	0.61	1.00
Social stress × PRS × group							0.68 (2)	0.71	1.00

*Note:* adj. *β*, standardized regression coefficients [continuous independent variables were standardized (mean = 0, s.d. = 1) for interpreting significant three-way interaction terms and examining the difference in associations between high (mean + 1 s.d.), and low (mean − 1 s.d.) PRS within and across groups (cases, siblings, controls)]; *p*_FWE_, family-wise error-corrected *p* values were computed by multiplying the unadjusted *p* value by the total number of tests (i.e. 4 stress measures × 3 outcomes = 12); CI, confidence interval; df, degrees of freedom; LR, likelihood ratio; s.d., standard deviation.

a Adjusted for age, gender and IQ.

b Difference in associations between those with high *v*. low PRS.

**Table 5.** Associations of momentary stress with psychotic experiences in cases, siblings and controls^a^

	Cases		Siblings		Controls		LR test for interaction^b^		
	adj. *β* (95% CI)	*p*	adj. *β* (95% CI)	*p*	adj. *β* (95% CI)	*p*	χ^2^ (df)	*p*	*p*_FWE_
Composite stress × PRS × group							19.70 (2)	<0.001	0.001
Level of PRS									
High	0.03 (−0.02 to 0.08)	0.26	−0.14 (−0.25 to 0.02)	0.02	0.05 (0.002 to 0.10)	0.04			
Low	0.06 (−0.01 to 0.12)	0.07	0.10 (0.01 to 0.20)	0.03	0.02 (−0.03 to 0.06)	0.45			
High *v*. low	−0.03 (−0.11 to 0.05)	0.48	−0.24 (−0.40 to −0.08)	0.003	0.03 (−0.03 to 0.09)	0.29			
Event-related stress × PRS × group							0.15 (2)	0.93	1.00
Activity-related stress × PRS × group							22.07 (2)	<0.001	<0.001
Level of PRS									
High	0.05 (−0.01 to 0.10)	0.08	−0.11 (−0.196 to −0.020)	0.02	0.12 (0.06 to 0.18)	<0.001			
Low	0.05 (−0.02 to 0.12)	0.13	0.08 (0.004 to 0.148)	0.04	0.02 (−0.03 to 0.07)	0.52			
High *v*. low	−0.01 (−0.09 to 0.08)	0.92	−0.18 (−0.304 to −0.064)	0.003	0.10 (0.03 to 0.17)	0.005			
Social stress × PRS × group							17.89 (2)	<0.001	0.001
Level of PRS									
High	0.02 (−0.03 to 0.07)	0.40	−0.12 (−0.25 to 0.02)	0.08	0.04 (−0.004 to 0.09)	0.07			
Low	0.02 (−0.04 to 0.08)	0.47	0.13 (0.03 to 0.24)	0.01	−0.003 (−0.04 to 0.04)	0.89			
High *v*. low	−0.001 (−0.08 to 0.08)	0.97	−0.25 (−0.44 to −0.07)	0.006	0.05 (−0.01 to 0.10)	0.13			

*Note:* adj. *β*, standardized regression coefficients [continuous independent variables were standardized (mean = 0, s.d. = 1) for interpreting significant three-way interaction terms and examining the difference in associations between high (mean + 1 s.d.), and low (mean − 1 s.d.) PRS within and across groups (cases, siblings, controls)]; *p*_FWE_, family-wise error-corrected *p* values were computed by multiplying the unadjusted *p* value by the total number of tests (i.e. 4 stress measures × 3 outcomes = 12); CI, confidence interval; df, degrees of freedom; LR, likelihood ratio; s.d., standard deviation.

a Adjusted for age, gender and IQ.

b Difference in associations between those with high *v*. low PRS.

**Table:** 

Delta high *v*. low PRS across groups	Cases *v*. controls		Cases *v*. siblings		Siblings *v*. controls	
	adj. *β* (95% CI)	*p*	adj. *β* (95% CI)	*p*	adj. *β* (*95% CI*)	*p*
Composite stress	−0.04 (−0.11 to 0.02)	0.19	0.12 (0.05 to 0.20)	0.001	−0.16 (−0.23 to −0.09)	0.001
Activity-related stress	−0.07 (−0.13 to −0.01)	0.02	0.08 (0.02 to 0.15)	0.01	−0.15 (−0.22 to −0.09)	<0.001
Social stress	−0.04 (−0.11 to 0.03)	0.24	0.13 (0.05 to 0.26)	0.001	−0.17 (−0.26 to −0.09)	<0.001

### Within-group comparisons (H1)

While there were no differences in the magnitude of associations of momentary stress with psychotic experiences within cases with high *v.* low level of PRS, this was the case for siblings and controls (see Table 5). In contrast to our hypothesis, there was a weaker association of composite momentary stress (*adj.β*_high *v*. low_ = −0.24, 95% CI −0.34 to −0.08, *p* = 0.003), activity-related stress (*adj.β*_high *v*. low_ = −0.18, 95% CI −0.30 to −0.06, *p* = 0.003) and social stress (*adj.β*_high *v*. low_ = −0.25, 95% CI −0.44 to −0.07, *p* = 0.006) with psychotic experiences in siblings with high PRS compared to siblings with low PRS. By contrast, in controls with high PRS, activity-related stress was associated with more intense psychotic experiences (*adj.β*_high *v*. low_ = 0.10, 95% CI 0.03–0.17, *p* = 0.005), than in controls with low PRS. The associations of social and event-related stress with psychotic experiences did not vary by PRS in controls.

### Between-group comparisons (H2)

When we examined whether PRS impacts differently on stress reactivity across groups based on differences in the magnitude of associations of momentary stress with psychotic experiences between those with high *v.* low PRS across groups (see Table 5), we observed consistent differences across siblings and controls as well as cases and siblings, but less consistent across cases and controls. We found evidence that the difference in associations of activity-related stress with psychotic experiences between those with high *v.* low PRS across groups was greatest in siblings *v.* controls (*adj.β*_delta high *v*. low_ = −0.15, 95% CI −0.22 to −0.09, *p* < 0.001) followed by cases *v.* siblings (*adj.β*_delta high *v*. low_ = 0.08, 95% CI 0.02–0.15, *p* = 0.01) and cases *v.* controls (*adj.β*_delta high *v*. low_ = 0.07, 95% CI −0.13 to −0.01, *p* = 0.02). We observed the greatest differences in associations of social stress and (iii) psychotic experiences between those with high *v.* low PRS across groups in siblings *v.* controls (*adj.β*_delta high *v*. low_ = −0.17, 95% CI −0.26 to −0.09, *p* < 0.001), followed by cases *v.* siblings (*adj.β*_delta high *v*. low_ = 0.13, 95% CI 0.05–0.26, *p* = 0.001). A similar pattern of findings emerged for differences in associations of composite momentary stress and psychotic experiences between individuals with high *v.* low PRS across groups. There was evidence that the difference in psychotic reactivity to composite momentary stress between those with high *v.* low PRS varied across siblings and controls (*adj.β*_delta high *v*. low_ = −0.16, 95% CI −0.24 to −0.09, *p* = 0.001) as well as cases and siblings (*adj.β*_delta high *v*. low_ = 0.12, 95% CI 0.05–0.19, *p* = 0.001).

## Discussion

### Principal findings

The current study is the first to investigate whether momentary stress reactivity is modified by PRS in cases with non-affective psychotic disorder, siblings of cases and controls. In contrast to our hypotheses (H1, H2), we found no evidence that associations of momentary stress with (i) negative affect and (ii) positive affect were modified by PRS and group. Further, the association between momentary stress and (iii) psychotic experiences was modified by PRS within siblings and controls, but not in cases. There was strong evidence that, in contrast to our first hypothesis, siblings with high PRS reported less intense psychotic experiences in response to momentary stress compared to siblings with low PRS. However, consistent with the first hypothesis, the opposite held true within controls, as individuals in this group with high PRS showed more intense psychotic experiences in response to stress compared to those with low PRS. We further found that differences in psychotic reactivity to momentary stress between high *v.* low PRS varied across groups, but these differences were not consistent with those posited in H2.

### Methodological considerations

The current findings should be interpreted in the light of some limitations. First, although numerous twin and family studies have suggested a high heritability for psychosis (Guloksuz et al., 2019; Islam et al., 2017; Sullivan, Kendler, & Neale, 2003), to date, the variance explained by PRS in molecular genetic studies remains limited and it is assumed that PRS only represent a part of the genetic contributions (Wray et al., 2021). In addition, evidence for an association with psychotic symptoms in the general population remains inconsistent. While some studies have reported an association between PRS and self-reported psychotic experiences in adolescents (Pain et al., 2018), no evidence for such an association has been found in the adult general population (Marsman et al., 2020; Mistry, Harrison, Smith, Escott-Price, & Zammit, 2018). Further, evidence for an association of PRS with negative symptoms remains equivocal in non-clinical populations (Mistry et al., 2018). However, in clinical populations, higher negative symptoms were associated with increased PRS (Bigdeli, Peterson, Docherty, Kendler, & Fanous, 2019; Mistry et al., 2018; Ruderfer & Psychiatric Genomics Consortium Bipolar/Schizophrenia Working Group, 2019). Moreover, Allardyce et al. (2018) showed a polygenic-risk gradient across schizophrenia and bipolar disorder that increased as levels of psychotic symptoms increased. This may suggest that PRS rather represents a genetic marker for negative symptoms in schizophrenia. In the present study, the ESM psychosis measure primarily assessed positive but not negative symptoms of schizophrenia, which may explain in part the inconsistent pattern of findings observed here. In addition, internal consistency of ESM measures for negative affect and psychotic experiences were moderate, which underlines the importance of further psychometric evaluation and validation of ESM measures.

Second, the PRS as calculated in the current study assumed additive effects of individual risk alleles, which reflects a rather simple genetic model. Complex higher-order interactive associations between risk alleles were not accounted for. This may in part explain why we did not find evidence of effect modification by PRS in cases.

Third, in line with previous research (Pries et al., 2020; Rauschenberg, van Os, Goedhart, Schieveld, & Reininghaus, 2021c), we used a composite stress measure and adjusted for multiple testing to minimize the potential impact of type I error rate. However, sample size and number of ESM observations were fairly small and, hence, may have provided limited statistical power for detecting three-way interaction effects. Hence, careful replication of our findings is required before firm conclusions can be drawn. Another methodological limitation is that other environmental factors such as childhood trauma (Lardinois, Lataster, Mengelers, Van Os, & Myin-Germeys, 2011; Rauschenberg et al., 2017; Reininghaus et al., 2016b) or bullying experiences (Rauschenberg et al., 2021c) have been shown to impact stress reactivity, but were not included in the current analysis given the limited sample size and number of ESM observations and, thus, confounding by, or further interaction with, these factors were not taken into account.

Last, the distribution of ESM data was skewed, which violates the assumption of normally distributed residuals in linear mixed models. However, when we fitted multilevel tobit regression models in a sensitivity analysis to assess how this may have impacted our findings, these remained largely unchanged. Also, cases completed fewer ESM assessments than siblings and controls, but this did not seem to be accounted for by interference of these assessments with their daily life.

### Comparison with previous research

To our knowledge, the present study is the first to investigate PRS and ESM data of a clinical sample. In many previous studies, genetic risk of psychosis has been approximated by family history, although this of course does not determine onset of the disorder (Lu et al., 2018). Myin-Germeys et al. (2001) previously observed that relatives compared to controls reported increased negative affect and psychotic experiences in response to momentary stress. Furthermore, stress sensitization has been postulated to comprise an important mechanism in pathways to psychosis (Collip et al., 2008; Myin-Germeys & van Os, 2007). We aimed to replicate and extend these findings and underpin the proposed aetiological model.

In the current study, we found that healthy individuals with higher polygenic risk for psychosis responded with more intense psychotic experiences to daily life stress compared to those with low polygenic risk. In line with this, psychotic reactivity to momentary stress has been reported to be modified by high polygenic risk (and exposure to childhood trauma) in healthy controls (Pries et al., 2020), which, in line with the stress sensitization model suggests that polygenic risk sensitizes healthy individuals to the psychosis-inducing effects of minor stressors in daily life. However, in contrast to this hypothesis, our results suggest no evidence of stress reactivity to be modified by polygenic risk in individuals with enduring psychotic disorder. One plausible explanation for this finding may be that the psychosis-inducing effects of minor stressors in daily life may particularly operate in individuals with high PRS primarily prior to onset, in the early stages of psychosis, and attenuate over time due to the effects of illness chronicity and exposure to antipsychotic medication (van der Steen et al., 2017).

Furthermore, in contrast to our hypotheses, siblings with high PRS appeared to be resilient to the exposure of stress as they reported less intense psychotic experiences in response to momentary stress. In the present study, siblings largely grew up in a shared environment with cases, which may have substantially contributed to familial liability. Having a close relative coping with psychotic experiences may affect individuals’ interpretation of their own experiences. As families experiencing mental health-related problems have more exposure to mental health-related information and mental health services, this may increase health literacy (Hurley, Swann, Allen, Ferguson, & Vella, 2020). Thus, siblings may better recognize early warning signs or show health-promoting behaviour when distressed. A high PRS for schizophrenia in relatives indicated an increased polygenic risk in individuals with increased familial liability to psychosis. There is evidence from previous research that PRS is higher in relatives than controls (van Os et al., 2020; van Os et al., 2017b), though, notably, in the present study a high PRS in relatives reflected a low PRS in cases of our sample. The marginal difference in PRS between siblings and controls in the present study may result from selecting the specific analytic sample used for the current analysis. Another explanation for the finding that stress reactivity is reduced in siblings may point to a genetic resilience factor that may have mitigated polygenic and environmental vulnerability. In fact, Hess, Tylee, Mattheisen, Borglum, and Glatt (2019) proposed a polygenic resilience score for schizophrenia, i.e. a heritable gene variation that reduces the penetrance of risk loci. To this end, they identified healthy relatives with high PRS for psychosis and compared this sample to risk-matched cases showing that the resilience score increases in unaffected individuals as their PRS increases. The specific role of buffering or protective factors in pathways to psychosis such as a polygenic resilience score for schizophrenia or a resilience-enhancing social environment (Gayer-Anderson & Morgan, 2013), and their impact on targetable mechanisms needs to be elucidated further as a basis for improving prevention (Rauschenberg et al., 2021a, 2021b; Reininghaus et al., 2016a), treatment (Schick et al., 2021; van Aubel et al., 2020) and, ultimately, outcomes of psychosis.

## Conclusion

In contrast to previous propositions, the current work provided no evidence that polygenic risk impacts reactivity to minor stressors in daily life in individuals with enduring non-affective psychotic disorder and prolonged exposure to antipsychotic medication. Our findings tentatively suggest that polygenic risk may operate in different ways than previously assumed and amplify stress reactivity in unaffected individuals but take on the role of a resilience factor in relatives by attenuating their stress reactivity. Stress reactivity may reflect a putative mechanism underlying polygenic risk and resilience to psychosis. Targeting this putative mechanism in individuals' daily life through novel ecological momentary interventions as experimental manipulation method is an important next step (Reininghaus et al., 2016a). This not only promises to further our understanding of how this mechanism impacts individuals with varying levels of risk but will also pave new ways to the prevention of, and treatment for, psychosis.

## Supplementary material

For supplementary material accompanying this paper visit https://doi.org/10.1017/S0033291721004761. click here to view supplementary material
