# Supplementary material for: Polygenic risk, familial liability and stress reactivity in psychosis: an experience sampling study
Source: Psychol Med. 2022 Jan 7;53(7):2798–807. doi: 10.1017/S0033291721004761 (PMC10235643; doi:10.1017/S0033291721004761)
Supplement: Supplementary file 1 [file S0033291721004761sup001.docx]

**Supplementary material**

1. **Genotyping, imputation and polygenic risk score computation**

Genotype data for 2,812 individuals was generated on a customized Illumina, IPMCN array with 570,038 SNPs. This chip contains ~250k common SNPs, 250K Exome chip variants (rare, exomic, nonsynonymous, MAF < 1%), and ~50K psychiatric-related variants. Quality control (QC) procedures were performed using PLINK v1.9 (Purcell *et al.*, 2007). SNPs and samples with call rates below 95% and 98%, respectively, were removed. A strict SNP QC only for subsequent sample quality control steps was conducted. This involved a minor allele frequency (MAF) threshold>10% and a Hardy-Weinberg equilibrium (HWE) p-value>1e-05, followed by linkage disequilibrium (LD) based SNP pruning (R2<0.2). This resulted in ~58K SNPs to assess sex errors, heterozygosity (F<3 standard deviation (S.D.)), homozygosity (F>3 S.D.) and relatedness by pairwise identity by descent (IBD) values. Duplicate samples (pihat > 0.8) were removed and remaining pairs were manually checked since this dataset contains family members. After removing failing samples, a regular SNP QC was performed (SNP call rate>98%, HWE p>1e-06, MAF>1%). After multidimensional scaling (MDS) clustering with Hapmap Phase 3 individuals to check ethnicity, samples that deviated more than 3 standard deviations from our dataset were removed (n=91). In addition, first 20 genetic Principal Components (PCs) of passed quality controlled samples were generated using the strict SNP QC list by EIGENSTRAT (Price *et al.*, 2006). Next, strand ambiguous SNPs and duplicate SNPs were removed. Mendelian errors were set to missing followed by another missingness check (2% threshold) for samples (n=8) and SNPs, and SNPs with a differential missingness between cases and controls were removed. In total, 2,505 individuals and 275,021 SNPs passed these abovementioned QC steps.

SNPs were imputed on the Michigan server (Das *et al.*, 2016) using the HRC r1.1 2016 reference panel with European samples after phasing with Eagle v2.3. Post-imputation QC involved removing SNPs with an Rsq info score<0.3, with a MAF<0.01, SNPs that had a discordant MAF compared to the reference panel, and strand ambiguous AT/CG SNPs and multi-allelic SNPs.

PRS for 2,505 samples were calculated using schizophrenia-associated alleles and effect sizes reported in the GWAS summary statistics from the Psychiatric genetics consortium schizophrenia working group freeze 2. To prevent potential overlap in study population to impact our results, all Dutch and Belgian individuals had been excluded from the PGC-2 GWAS to allow unbiased PRS computation. Overlapping SNPs between the PGC-2 GWAS (training dataset), 1000 reference Genome (reference dataset), and our dataset (target dataset) were selected. Then, insertion or deletion, ambiguous SNPs, SNPs with minor allele frequency (MAF)<0.01, SNPs with imputation quality (R2)<0.8 in both training dataset and target dataset, and SNPs located in complex-LD regions (Price *et al.*, 2008) (supplementary Table S1) were excluded, leaving 2,950,238 SNPs. These SNPs were clumped in two rounds using PLINK; round 1 with the default parameters (physical distance threshold 250kb and LD threshold (R2) <0.5); round 2 with a physical distance threshold of 5,000kb and LD threshold (R2)< 0.2; resulting in 194,665 SNPs for PRS calculation. Odds ratios for autosomal SNPs reported in the schizophrenia summary statistics were log-converted to beta values. PRS were calculated using PLINK’s score function for 12 GWAS p-value thresholds (5 × 10^-8^, 5 × 10^-7^, 5 × 10^-6^, 5 × 10^-5^, 5 × 10^-4^, 5 × 10^-3^, 0.05, 0.1, 0.2, 0.3, 0.4 and 0.5). The PRS pt=0.05 explained most of the variance of SCZ case control status. Hence, we selected the PRS pt=0.05 as a proxy for the SCZ genetic profile score to perform the following regression analyses.

From 2505 individuals, we selected 706 SCZ cases and 368 non-relative healthy controls for the binomial logistic regression model. The model equation is

$$\log\left( \mathrm{SCZ}_{\mathrm{case}_{\mathrm{control}_{\mathrm{status}}}} \right)\sim age+sex+PC1+PC2+PC3+PRS$$

The explained variance is represented as Nagelkerke R^2^, see supplementary Figure S1.

**Table S1.** Removed complex-LD regions (build GRCh37) before PRS calculation.

| Chromosome | Base pair start | Base pair end |
| --- | --- | --- |
| 6 | 25392021 | 33392022 |
| 8 | 111930824 | 114930824 |
| 11 | 46043424 | 57243424 |
| 1 | 48287980 | 52287979 |
| 2 | 86088342 | 101041482 |
| 2 | 134666268 | 138166268 |
| 2 | 183174494 | 190174494 |
| 3 | 47524996 | 50024996 |
| 3 | 83417310 | 86917310 |
| 3 | 88917310 | 96017310 |
| 5 | 44464243 | 50464243 |
| 5 | 97972100 | 100472101 |
| 5 | 128972101 | 131972101 |
| 5 | 135472101 | 138472101 |
| 6 | 56892041 | 63942041 |
| 6 | 139958307 | 142458307 |
| 7 | 55225791 | 66555850 |
| 8 | 7962590 | 11962591 |
| 8 | 42880843 | 49837447 |
| 10 | 36959994 | 43679994 |
| 11 | 87860352 | 90860352 |
| 12 | 33108733 | 41713733 |
| 12 | 111037280 | 113537280 |
| 20 | 32536339 | 35066586 |

**Figure S1**. PRS explained variance of SCZ outcome in GROUP data.

Legend: P-value thresholds (P_T_) for SCZ SNPs are shown on the x axis, where the number of SNPs increases with a more lenient P_T_. Δ Explained variances (Nagelkerke *R^2^*, shown as %) of a generalized linear model including SZ-based PRS versus a baseline model without polygenic scores (blue bars) are shown for each P_T_. –Log_10_ P-values of Δ explained variance per P_T_ (red dots) represent P-values from the binomial logistic regression.

Note: SCZ, Schizophrenia;

**Table S2.** Sample characteristics of the GROUP sample with no ESM/ PRS data and comparison with analytic sample.

|  | | | Cases  (n=1022) | |  | Siblings  (n=977) |  | Controls  (n=511) |  | *Cases vs.  analytic cases* | | *Siblings vs.  analytic siblings* | | *Controls vs.  analytic controls* | |
| --- | --- | --- | --- | --- | --- | --- | --- | --- | --- | --- | --- | --- | --- | --- | --- |
|  | | | |  |  |  |  |  |  | adj. B  (95% CI) | p | adj. B  (95% CI) | p | adj. B  (95% CI) | p |
| Age (years), mean  (S.D.) | | | | 29.73 (8.04) |  | 30.05  (8.27) |  | 32.25 (10.63) |  | -3-56 (-5.23 – -1.90) | <.001 | -4.76 (-6.64 – -2.87) | <.001 | -7.70 (-10.35 – -5.05) | <.001 |
| Gender, n (%) | | | |  |  |  |  |  |  | -0.12 (-0.21 – - 0.03) | .007 | -0.08 (-0.20 – 0.03) | .15 | -0.22 (-0.34 – -0.10) | <.001 |
|  | Male | | | 789 (77.20) |  | 451 (46.16) |  | 249  (48.73) |  |  |  |  |  |  |  |
|  | Female | | | 233  (22,80) |  | 526  (53.84) |  | 259  (50.68) |  |  |  |  |  |  |  |
| IQ estimate, mean (S.D.) | | | | 95.74  (16.19) |  | 104.96 (16.40) |  | 110.95  (15.26) |  | -5.87 (-9.26 – -2.49) | <.001 | -5.14 (-8.82 – -1.46) | .006 | -3.16 (-6.87– 0.55) | .10 |
| Clinical characteristics, mean (S.D.) | | | |  |  |  |  |  |  |  |  |  |  |  |  |
|  | | Social functioning | | 112.74  (8. 89) |  | 122.43 (6.25) |  | 124.47  (5.09) |  | -1.45  (-3.32 – 0.42) | .13 | -0.88 (-2.32 – 0.56) | .23 | 0.02 (-1.25 – 1.27) | .98 |
|  | | PANSS positive | | 13.31  (5.80) |  | 7.37 (1.42) |  | 7.27  (1.05) |  | 1.59 (0.40 – 2.79) | .009 | 009 (-0.25 – 0.42) | .61 | -0.0002 (-0.22 – 0.22) | .99 |
|  | | PANSS negative | | 14.36  (5.83) |  | 8.55 (1.60) |  | 8.28  (0.95) |  | 2.57 (1.38 – 3.76) | <.001 | 0.27 (-0.10 – 0.64) | .15 | 0.16 (-0.06 – 0.37) | .15 |

*Note:* PRS, Polygenic risk score; PANSS, Positive and Negative Syndrome Scale; S.D., standard deviation; vs., versus; CI, confidence interval.

**Table S3.** Tobit regression of momentary stress with negative affect, positive affect and psychotic experiences in cases, relatives and controls ^a^.

|  | | | | Cases | | Siblings | | | | Controls | | | LR Test for interaction ^b^ | |  |
| --- | --- | --- | --- | --- | --- | --- | --- | --- | --- | --- | --- | --- | --- | --- | --- |
|  |  |  |  | adj. *β*  (95% CI) | *p* |  | adj. *β*  (95% CI) | *p* |  | adj. *β*  (95% CI) | *p* |  | *χ*^2^  (df) | *p* | *p_FWE_* |
| Outcome: Negative affect | | | | | | | | | | | | | | |  |
| Composite stress × PRS × group | | | |  |  |  |  |  |  |  |  |  | 9.18  (2) | .01 | .12 |
| Event-related stress × PRS × group | | | |  |  |  |  |  |  |  |  |  | 1.78  (2) | .41 | 1.00 |
| Activity-related stress × PRS × group | | | |  |  |  |  |  |  |  |  |  | 15.41  (2) | .0005 | .006 |
|  | Level of PRS | | |  |  |  |  |  |  |  |  |  |  |  |  |
|  |  | High | | 0.36  (0.27 – 0.44) | <.001 |  | 0.15  (-0.07 – 0.36) | .18 |  | 0.52  (0.36– 0.68) | <.001 |  |  |  |  |
|  |  | Low | | 0.35  (0.25 – 0.45) | <.001 |  | 0.67  (0.46 – 0.88) | <.001 |  | 0.31  (0.13 – 0.49) | .001 |  |  |  |  |
|  |  | High vs. low | | 0.01  (-0.12 – 0.14) | .88 |  | -0.52  (-0.86 – -0.19) | .002 |  | 0.21  (-0.01 – 0.43) | .07 |  |  |  |  |
| Social stress × PRS × group | | | |  |  |  |  |  |  |  |  |  | 6.93  (2) | .03 | .36 |
| Outcome: Positive affect | | | | | | | | | | | | | | |  |
| Composite stress × PRS × group | | | |  |  |  |  |  |  |  |  |  | 0.59  (2) | .74 |  |
| Event-related stress × PRS × group | | | |  |  |  |  |  |  |  |  |  | 0.03  (2) | .98 |  |
| Activity-related stress × PRS × group | | | |  |  |  |  |  |  |  |  |  | 0.74  (2) | .69 |  |
| Social stress x PRS x group | | | |  |  |  |  |  |  |  |  |  | 0.57  (2) | .75 |  |
| Outcome: Psychotic experiences | | | | | | | | | | | | | | |  |
| Composite stress x PRS x group | | | |  |  |  |  |  |  |  |  |  | 11.55  (2) | .003 | .036 |
|  | Level of PRS | | |  |  |  |  |  |  |  |  |  |  |  |  |
|  |  | | High | 0.12  (0.01 – 0.23) | .04 |  | -0.69  (-1.61 - 0.23) | .14 |  | 0.10  (-0.39 – 0.59) | .69 |  |  |  |  |
|  |  | | Low | 0.18  (0.06 – 0.30) | .003 |  | 0.77  (0.05 – 1.50) | .04 |  | 0.17  (-0.45 – 0.80) | .59 |  |  |  |  |
|  |  | | High vs. low | -0.06  (-0.23– 0.10) | .44 |  | -1.46  (-2.70 – -0.22) | .02 |  | -0.07  (-0.79 – 0.65) | .85 |  |  |  |  |
| Event-related stress × PRS × group | | | |  |  |  |  |  |  |  |  |  | 1.0  (2) | .61 | 1.00 |
| Activity-related stress × PRS × group | | | |  |  |  |  |  |  |  |  |  | 19.07  (2) | <.001 | <.001 |
|  | Level of PRS | | |  |  |  |  |  |  |  |  |  |  |  |  |
|  |  | | High | 0.18  (0.08 – 0.29) | .001 |  | - |  |  | 0.55  (0.10 – 1.0) | .02 |  |  |  |  |
|  |  | | Low | 0.23  (0.11 – 0.34) | <.001 |  |  |  |  | -0.28  (-0.91 – 0.34) | .38 |  |  |  |  |
|  |  | | High vs. low | -0.04  (-0.09 – 0.11) | .57 |  |  |  |  | 0.83  (0.09 – 1.57) | .03 |  |  |  |  |
| Social stress × PRS × group | | | |  |  |  |  |  |  |  |  |  | 7.29  (2) | .03 | .36 |

*Note:* adj. β, Standardized regression coefficients [continuous independent variables were standardized (mean = 0, S.D. = 1) for interpreting significant three-way interaction terms and examining the difference in associations between high (mean + 1 S.D.), and low (mean− 1 S.D.) PRS within and across groups (cases, relatives, controls)]; p_FWE_, family-wise error-corrected p-values were computed by multiplying the unadjusted p-value by the total number of tests (i.e. 4 stress measures x 3 outcomes = 12); CI, confidence interval; df, degrees of freedom; LR, likelihood ratio; S.D., standard deviation.

^a^ Adjusted for age, gender and IQ.

^b^ Difference in associations between those with high vs. low PRS:

|  |  | *Cases vs. controls* | | *Cases vs. siblings* | | *Siblings vs. controls* | |
| --- | --- | --- | --- | --- | --- | --- | --- |
|  |  | adj. *β* (95% CI) | *p* | adj. *β* (95% CI) | *p* | *adj. β (95% CI)* | *p* |
| Outcome: Negative affect | | | | | | | |
| Delta high vs. low PRS across groups | |  |  |  |  |  |  |
|  | Activity-related stress | -0.10 (-0.12 – 0.02) | .11 | 0.24 (0.087 – 0.391) | .002 | -0.34(-0.507 – -0.168) | <.001 |
| Outcome: Psychotic experiences | | | | | | | |
| Delta high vs. low PRS across groups | |  |  |  |  |  |  |
|  | Composite stress | -0.02 (-0.33 – 0.29) | .90 | 0.56 (0.22 – 0.89) | .001 | -0.58 (-0.98 – -0.18) | .004 |
|  | Activity-related stress | -0.31 (-0.54 – -0.07) | .01 | 0.40 (0.15 – 0.65) | .002 | -0.70 (-1.02 – -0.39) | <.001 |

References

**Das, S., Forer, L., Schönherr, S., Sidore, C., Locke, A. E., Kwong, A., Vrieze, S. I., Chew, E. Y., Levy, S., McGue, M., Schlessinger, D., Stambolian, D., Loh, P.-R., Iacono, W. G., Swaroop, A., Scott, L. J., Cucca, F., Kronenberg, F., Boehnke, M., Abecasis, G. R. & Fuchsberger, C.** (2016). Next-generation genotype imputation service and methods. *Nature Genetics* **48**, 1284-1287.

**Price, A. L., Patterson, N. J., Plenge, R. M., Weinblatt, M. E., Shadick, N. A. & Reich, D.** (2006). Principal components analysis corrects for stratification in genome-wide association studies. *Nat Genet* **38**, 904-9.

**Price, A. L., Weale, M. E., Patterson, N., Myers, S. R., Need, A. C., Shianna, K. V., Ge, D., Rotter, J. I., Torres, E., Taylor, K. D., Goldstein, D. B. & Reich, D.** (2008). Long-range LD can confound genome scans in admixed populations. *Am J Hum Genet* **83**, 132-5; author reply 135-9.

**Purcell, S., Neale, B., Todd-Brown, K., Thomas, L., Ferreira, M. A., Bender, D., Maller, J., Sklar, P., de Bakker, P. I., Daly, M. J. & Sham, P. C.** (2007). PLINK: a tool set for whole-genome association and population-based linkage analyses. *Am J Hum Genet* **81**, 559-75.
